# Should responsibility be used as a tiebreaker in allocation of deceased donor organs for patients suffering from alcohol-related end-stage liver disease?

**DOI:** 10.1007/s11019-023-10141-3

**Published:** 2023-02-13

**Authors:** Diehua Hu, Nadia Primc

**Affiliations:** 1 Department of Philosophy, Central South University, 410075 Changsha, China; 2 Institute of History and Ethics of Medicine, Medical Department, Heidelberg University, Im Neuenheimer Feld 327, 69120 Heidelberg, Germany

**Keywords:** Alcohol-related end stage liver disease (ARESLD), Liver transplantation, Organ allocation, Responsibility, Tiebreaker, Ethics

## Abstract

There is a long-standing debate concerning the eligibility of patients suffering from alcohol-related end-stage liver disease (ARESLD) for deceased donor liver transplantation. The question of retrospective and/or prospective responsibility has been at the center of the ethical discussion. Several authors argue that these patients should at least be regarded as partly responsible for their ARESLD. At the same time, the arguments for retrospective and/or prospective responsibility have been strongly criticized, such that no consensus has been reached. A third option was proposed as a form of compromise, namely that responsibility should only be used as a tiebreaker in liver allocation. The present study provides an ethical investigation of this third option. First, we will provide an overview of the main arguments that have been offered for and against the use of responsibility as an allocation criterion. Second, we will explore the concept of responsibility as a tiebreaker in detail and discuss several types of situations, in which responsibility could be used as a tiebreaker, as well as the main ethical challenges associated with them. As we will show, an ethical justified use of responsibility as a tiebreaker is limited to a very restricted number of cases and is associated with a number of ethical concerns. For this reason, waiting time should be preferred as a tiebreaker in liver allocation, even though the criterion of waiting time, too, raises a number of equity-related concerns.

## Introduction

Under the current and potentially increasing conditions of scarcity of donor organs, ethically challenging decisions have to be made regarding the allocation of deceased donor organs. Since the 1980s, ethical concerns have been raised regarding the eligibility of patients suffering from alcohol-related end-stage liver disease (ARESLD) for deceased donor liver transplants (Starzl et al. 1988; Im et al. 2019). Different framings and lines of justification have been offered to justify lower priority of these patients and/or the implementation of a six-month abstinence period before these patients are granted access to the waiting list for a donor organ (Notini et al. 2019; Primc 2020a). In the ethical debate, the question of responsibility has been at the center of the controversy (Notini et al. 2019). Some scholars support the view that alcoholics should be held at least partly responsible for their behavior in form of persistent and excessive alcohol consumption that leads to end-stage liver disease (ELSD). They argue that although alcoholism is a disease, individuals should still be regarded as autonomous beings that have (or had) several options at their disposition to control their actions and choices. As a result, proponents of the responsibility argument argue that, given the extreme scarcity of organs, it is justified and fair to attribute lower priority to patients with ARESLD than patients suffering from liver diseases that are not directly linked to an unhealthy and supposedly irresponsible lifestyle.

Conversely, several authors claim that responsibility should not be used as a criterion for the allocation of scarce medical resources for a number of reasons. Two points of criticism were given particular attention, namely (1) empirical-practical difficulties in determining the varying degree of responsibility of individual patients for their ARESLD that raise questions of equity and fairness, (2) concerns regarding the negative impact that the assessment of patients’ responsibility for their liver failure could have on the relationship between doctors and their patients, on the willingness of patients to openly share health-related information with their doctors, as well as on the stigmatization of patients suffering from ARESLD (Glannon 2009; Ho 2008; Notini et al. 2019; Thornton 2009; Veatch and Ross 2015).

Both parties generally concede that the opposing side raises some valuable points, but disagree on the relevance of these objections, so that despite long-standing debates, no agreement is in sight (Sharkey and Gillam 2010). In view of this unresolved situation, a third option was offered as a form of compromise, namely that responsibility should only be used as a tiebreaker in liver allocation (e.g. Thornton 2009). This means that personal responsibility is not used as a full-fledged allocation criterion alongside generally accepted criteria such as urgency or prospects of success, but will only be considered in cases in which an “allocation stalemate” occurs. The latter refers to situations in which the generally accepted and less controversial allocation criteria (e.g. urgency, prospects for success) are not sufficient to make a decision regarding the allocation of a particular organ, i.e. two (or even more) patients are regarded as equally suitable and show similar degrees of urgency, prospects for success, and possible other criteria that are used as allocation criteria in a specific allocation system.^1^ It has been argued that although responsibility may face some serious challenges if used as a full criterion, it could at least be used as a tiebreaker in these situations. This third option has been offered as a possibility to reconcile the debate over responsibility as a criterion for liver allocation.

Although this third option has been occasionally mentioned in the debate (e.g. Brudney 2007, Glannon 2009, Thornton 2009; Veatch and Ross 2015), to the best of our knowledge, no scholar has examined this option in more detail. The present study provides an ethical investigation of this third option, i.e. the use of responsibility as a tiebreaker in liver allocation. First, we will provide an overview of the main arguments that have been offered for and against the use of responsibility as an allocation criterion (Sect. 2). We will not give an extensive account of the ethical debate (overviews can, for example, be found in Sharkey and Gillam 2010 and Notini et al. 2019) but focus on those groups of arguments that will play an essential role in our subsequent discussion of the tiebreaker option. Second, we will explore the concept of (partial) responsibility as a tiebreaker in more detail and discuss several types of situations, in which responsibility could be used as a tiebreaker, as well as the main ethical challenges associated with them (Sect. 3). As we will show, an ethical justified use of responsibility as a tiebreaker is limited to a very restricted number of cases and comes with several potential negative consequences. For this reason, alternative tiebreakers, such as the waiting time, should be preferred, even though the criterion of waiting time is also associated with a number of equity-related concerns.

## Partial responsibility as a justification for lower priority of patients suffering from ARESLD

Excessive and harmful consumption of alcohol has for a long time in history been regarded as a form of vice in society as well as medicine, for which individuals could in principle be held responsible. It was only in the second half of the 20th Century, that the “disease concept of alcoholism” became the predominant approach in medicine (Jellinek 1960). In the current eleventh revision of the International Classification of Diseases (ICD-11), alcohol dependence and harmful alcohol consumption form part of the section on “disorders due to substance use and addictive behaviours” (Saunders et al. 2019). Given the broad consensus that alcohol dependence is an addictive disease that can go along with an impaired control over alcohol use (Saunders et al. 2019), most researchers agree that patients suffering from ARESLD cannot be regarded as fully responsible for their addictive behavior. Hence, the debate on lower priority for patients suffering from ARESLD has focused on arguments for partial responsibility. The following discussion will only refer to the postulation of a partial responsibility, since the assumption of a complete responsibility does not seem tenable.

Proponents of the partial responsibility argument insist that although genetic, socio-economic and cultural factors may play a significant role in the development of harmful drinking patterns and alcohol dependence, patients still have (or had) enough autonomy to control part of their choices. Hence, it should be regarded as justified and fair to give patients suffering from ARESLD under the current conditions of extreme scarcity of donor organs lower priority than patients who developed a form of ESLD through no fault of their own. For example, Veatch argues that denying patients with ARESLD any responsibility for the development of their liver disease would amount to a “tragic view of human behavior that, carried to the extreme, makes all ethics impossible” (Veatch and Ross 2015, p. 309). Although genetic, socio-economic and cultural factors may have an impact on the development of addictive behavior, we have to assume that the behavior of patients suffering from ARESLD is at least to “some degree, free and open to choice” (Veatch and Ross 2015, S. 309), as any other position would, in Veatch’s opinion, entail a deterministic view of human nature (Veatch and Ross 2015, p. 309). Hence, patients can (and should) be held at least partly responsible for the choices they made.

Based on the assumption that patients suffering from ARESLD have at least some degree of control over their behavior, researchers discuss two types of responsibility, (1) partial retrospective responsibility for developing an addiction and the decision to start drinking in the first place (2) partial prospective responsibility for not seeking treatment to prevent further deterioration of their health status and addictive behavior (Glannon 1998; Notini et al. 2019; Thornton 2009). Both types of partial responsibility will be briefly discussed.

### (Partial) retrospective responsibility: responsibility for the development of ARESLD

As a prominent proponent of retrospective responsibility, Glannon (1998) stresses that ARESLD is a preventable outcome as people, in general, have a certain degree of causal control over the factors that lead to the development of their addictive behavior and subsequent liver failure. Glannon cites several conditions that must be met in order to attribute causal responsibility to persons for the occurrence of an event, i.e. that persons must not be coerced by external or internal factors, that they should be capable of reflective self-control and have knowledge on the consequences of an unhealthy life style as well as on the consequences this could have on their access to the life-saving resource of donor organs (Glannon 1998). These factors may vary in individual cases (f. ex. according to age, educational status, socioeconomic factors) such that the degree of retrospective responsibility in patients with ARESLD can vary too. That is why a detailed etiology or history of the disease needs to be established in order to assess the degree of causal control and responsibility in each individual case (Glannon 1998). But even in cases, in which individuals have, for example, a higher probability of developing an addiction due to some genetic predispositions, the genes do not determine the behavior of the individual and his/her decision to start drinking in the first place (Glannon 2009). If a person fulfils all these conditions for causal responsibility and still voluntarily decides to engage in health-damaging behavior, it would be fair, according to the proponents of retrospective responsibility, to give this person lower priority than patients who had no causal control over the development of an ESLD and, hence, never an opportunity to influence the progression of their disease.

As this brief account shows, the argument for retrospective responsibility is restricted to a number of conditions. Not every patient is equally responsible for their ARESLD, especially if their addiction started at a very young age (Thornton 2009), or is strongly influenced by socioeconomic factors, or early role models that they encountered in their youth. In practice, it may be very challenging to determine the degree of responsibility and causal control that an individual had over the development of his/her addiction (Brudney 2007). These practical challenges have led some authors (who basically take a positive stance towards the responsibility argument) to restrict the use of retrospective responsibility as a distributive criterion to a few very specific cases, f. ex. to allocation decisions on the macro level of the health care system (e.g. in the determination of health care schemes, see Buyx 2008), to cases in which a re-transplantation because of ARESLD becomes necessary (Brudney 2007), to a rather modest negative factor in the allocation of organs (f. ex. by subtracting 1–2 points on the waiting list, Veatch and Ross 2015, p. 316), or to cases in which a tiebreaker is needed to make an allocation decision (e.g. Thornton 2009). It is the latter compromise, that will be discussed in more detail in the present investigation. The tiebreaker idea has been proposed by several proponents of the responsibility argument as a possibility to circumvent these difficulties in determining the level of responsibility.

### (Partial) prospective responsibility: responsibility for not seeking treatment

Given the difficulties in determining the degree of causal control and retrospective responsibility that a patient had over the development of his/her alcohol disorder, some scholars argue that although alcoholics cannot be easily held responsible for the development of their addiction, they are still responsible for not seeking treatment to prevent the deterioration of their health status that may ultimately result in ARESLD (Moss and Siegler 1991; Bailey et al. 2013). Retrospective responsibility refers to the responsibility “for becoming an alcoholic”, whereas prospective responsibility asks whether a patient can be held “responsible for remaining an alcoholic long enough to develop liver disease” (Ubel 1997, p. 343 f.). Prospective responsibility also includes patients’ responsibility to increase the efficiency of therapy by modifying their unhealthy lifestyles and optimizing their prospects for success (Feiring 2008). Nevertheless, a distinction should be made between the discussion surrounding prospective responsibility and arguments that want to exclude patients with an active addiction from transplantation because of allegedly low prospects of success. The former would allow to give patients a lower priority even if they had very good prospects of success and/or a certain period of alcohol abstinence.

The issue of prospective responsibility for seeking medical treatment points to an important challenge in the treatment and care of patients with alcohol disorders, namely that these patients have a very low treatment prevalence compared to other mental disorders (Kohn et al. 2004; Carvalho et al. 2019). Identification of patients suffering from alcohol dependence represents a major challenge in primary as well as specialized care (Rehm et al. 2015). Studies show that alcohol dependence is nowadays still associated with negative stereotypes (e.g. it is regarded as a vice, or weakness of character) and these negative stereotypes are known to represent important obstacles for patients to actively seek treatment for their alcohol disorder (Schomerus et al. 2014). Patients suffering from alcohol disorders represent for various reasons a group with a large, though unspecified treatment gap. This treatment gap increases the risk of liver disease progression, thereby posing a significant challenge for the continuing shortage of donor organs. Against this background, several authors claim that the willingness to seek treatment should be included in the allocation decisions, especially as such a policy could provide an important incentive to the larger population to refrain from or give up harmful habits (Brudney 2007, p. 44).

### Criticism and concerns about using (partial) responsibility as an allocation criterion in liver allocation

The proposal to include responsibility as a criterion in the allocation of donor organs has been criticized for several reasons. We will confine ourselves to the two most prominent groups of objections, that will also play a role in the subsequent discussion of the tiebreaker option, namely (1) that the already mentioned practical challenges in determining the level of control and responsibility are linked to fundamental problems of equity and fairness, and (2) that these challenges will have far-reaching negative effects, especially on the doctor-patient relationship and the stigmatization of patients suffering from ARESLD.

The first point of criticism relates to the assertion that patients still have a sufficient degree of control over their behavior such that they can be held retrospectively and/or prospectively responsible for their ARESLD. A number of difficulties were cited in opposition to this claim. Considering the concept of retrospective responsibility, the assertion has been questioned that individuals deliberately take the risk of developing an addiction. It is in most cases not at all a decision that is made in an informed and deliberate manner (Feiring 2008). Several external factors such as poverty, class, or early role models can have a strong impact on the drinking behavior of individuals. The influence of socioeconomic factors makes it difficult to determine whether and to what extent a patient’s irresponsible and risky behavior is a product of free and deliberate choice (Golan 2010). The concept of prospective responsibility faces some shortcomings, too. For example, many patients with alcohol disorders suffer from additional mental disorders such as personality disorders, schizophrenia, depression, and social phobia (Boden and Fergusson 2011; Fernandez-Montalvo et al. 2006; Lépine and Pélissolo 1998; Martens 2001; Soyka 2000), which can severely impair their capacity for voluntary control and, hence, their ability to actively and successfully fight their addiction. The critical role of neurological and biochemical factors in the etiology of alcoholism may severely impair patients’ control over their addictive behavior. All these factors can severely limit patients’ capacities to actively take responsibility for their decisions regarding initiation of treatment and compliance to treatment (DePergola 2018).

This non-exhaustive list of factors suggests that individual patients have varying degrees of (retrospective/prospective) responsibility for the development and progression of their ARESLD and that it is extremely difficult or even impossible to empirically determine what degree of (retrospective/prospective) responsibility should be ascribed to them (Sharkey and Gillam 2010; Notini et al. 2019). These empirical and epistemological difficulties lead to normative concerns: If the degree of responsibility cannot be adequately determined, it should not and cannot be used as a justification to give a whole group of patients lower priority in their access to a life-sustaining resource, let alone to exclude them. And even if we were in a position to empirically determine the individual degree of responsibility with sufficient precision, this would pose the challenge to decide in a reasoned manner what amount of lower priority and level of punishment should be regarded as appropriate for a given degree of retrospective and/or prospective responsibility (Thornton 2009, Veatch and Ross 2015, Zambrano 2016). As already mentioned above, the tiebreaker option has been specifically offered by proponents of the (partial) responsibility argument as a possibility to circumvent these difficulties in determining the level of responsibility. Whether this is an ethically justifiable solution will be discussed in Sect. 3.

Another closely related issue has been discussed under the heading of “moral luck”, which generally describes the phenomenon that factors beyond our control may partially determine our moral praiseworthiness or blameworthiness (Hartman 2017, p. 23). Some patients (e.g. women) develop ESLD much more quickly than other, who can consume the same amount of alcohol without putting themselves in a life-threatening situation. This raises questions of equity and justice, as one person is punished for risky behavior that has no consequences at all for another person, only because of some lucky circumstances that are beyond their control (Brudney 2007, Cohen and Benjamin 1991). The above-mentioned influence of genetic and socio-economic factors on the development of abusive and addictive drinking patterns has also been discussed under the topic of moral luck, especially as they can be present in different combinations in individual persons. All of these points have been cited as objections against the responsibility argument and, hence, as a justification of the general claim that any allocation that attributes lower priority to patients with ARESLD just because of their type of disease should be considered as unfair (Atterbury 1986; Flavin et al. 1988, Lucey and Beresford 1992, Prado et al. 2016).

A second major group of criticism raises concerns regarding some negative impacts that the assessment of retrospective and/or prospective responsibility would have, especially on the patient-doctor relationship. If the doctor has to judge the responsibility of his patients, this can negatively affect the trust relationship between doctor and patient (Ho 2008). Critics believe that doctors will be pushed into the role of judges (Sharkey and Gillam 2010). Out of fear of being disadvantaged, patients may feel compelled to withhold or misrepresent information about their illness. The distorted information makes it more challenging for the doctor to make appropriate and informed diagnoses and treatment recommendations (Ho 2008; Thornton 2009, Veatch and Ross 2015). Further negative consequences were mentioned in the debate, for example, that the verification and determination of the degree of responsibility represents an intensive and time-consuming process if it is to meet high standards of medical care (Martens 2001, Sharkey and Gillam 2010, Thornton 2009, Tonkens 2018). Furthermore, such a policy may reinforce the stigmatization of patients suffering from alcohol disorders, which in turn could increase the above-mentioned treatment gap (Benjamin 1997; Feiring 2008).

As this brief account shows,^2^ both sides have important arguments in their favor. The question of the use of partial responsibility as an allocation criterion remains a subject of debate, without any clear agreement in sight (Sharkey and Gillam 2010). It is against this background that the tiebreaker option was presented as a compromise that is able to combine the advantages and avoid the disadvantages of both positions. This third option will be the focus of the remaining sections.

## The third option: responsibility as a tiebreaker in liver allocation

Before engaging into an analysis of responsibility as a tiebreaker, the question arises as to why one should deal with this option in more detail, especially if one considers all the challenges mentioned in the previous section. The main reason is that ARESLD is not a rare disease. Rather, it is one of the most common diagnoses in patients with ESLD and it is expected by some scholars to become the leading cause of liver transplantation in the future (Testino 2017). It is the second leading cause of liver transplants in the United States of America (USA) and Europe (Giard et al. 2019; Mellinger and Volk 2018; Siddiqui and Carlton 2016). In 2019, the only more frequent diagnosis than ARESLD in the USA was “other/unknown”, which includes several different diagnoses, especially non-alcoholic steatohepatitis (Kwong et al. 2021). A recent study suggests that ARESLD has become the most common indication for liver transplant waitlisting among young adults (age 20–40) in the USA. The retrospective study of liver transplant listing from 2003 to 2018 shows that ARESLD appears to be the main reason for the increasing number of liver transplant listing in young adults, especially in young women (Philip et al. 2022). Hence, the patients suffering from ARESLD represent a major burden for the scarce resource of donor livers which can lead to increasing frustration in patients who suffer from liver failure through no fault of their own. The tiebreaker option is seen by some authors as a possibility to accommodate this sense of injustice, while still acknowledging the above-mentioned concerns about the use of responsibility as an allocation criterion and especially the fact that ARESLD is a disease.

### Definition and implicit conditions of the use of responsibility a tiebreaker

Using responsibility as a tiebreaker means that a history of alcohol abuse will only be considered in case that two (or more) candidates are deemed “equally suitable” for a donor liver. This corresponds to the understanding that patients suffering from ARESLD can only be held partially responsible for their illness, that it is almost impossible to empirically determine the degree of responsibility, and, hence, these patients should only be given lower priority in very specific cases. Within the framework of the tiebreaker option, no explicit distinction is usually made between the different types of responsibility, but rather both forms of responsibilities, however small they may be in individual cases, are seen as a sufficient justification to use responsibility as a tiebreaker.

However, a clear distinction needs to be made between the tiebreaker option and several other allocation strategies that are sometimes discussed together. For example, one of the procedures discussed by Veatch cannot, strictly speaking, be classified as a tiebreaker strategy. The Model for End-Stage Liver Disease (MELD) is used worldwide as a basis for liver allocation. It is a mathematical model that is used as a predictor of three-month waiting-list mortality and, hence, as a scale for the urgency of transplantation and severity of disease. In its basic form, the MELD score is based on serum creatinine, bilirubin, and international normalized ratio (a test that measures time for blood to clot) and ranges from 6 to 40 (Asrani and Kamath 2015). As Veatch points out “a history of alcoholism could be reflected by subtracting a point or two. The number would be set in proportion to how important the voluntary component seemed to be” (Veatch and Ross 2015, p. 316). In such a case, responsibility is not used as a tiebreaker, but rather as a full, albeit rather weak, allocation criterion that is applied to all ARESLD patients.^3^ This is also the case for the widely applied six-month abstinence period, which requires patients to have a six-month abstinence period before they can be placed on the transplant waiting list. Although different lines of justification were offered for the implementation of the six-month abstinence period (e.g. Primc 2020a), it is used as a general criterion for inclusion on the waiting list and not just as a tiebreaker.

The concept of responsibility as a tiebreaker restricts its use to situations in which two candidates are considered as equally appropriate recipients for a donor liver, e.g. they show similar prospects of success and degrees of urgency (e.g. Glannon 2009, p. 24, Veatch and Ross 2015, p. 316). The proponents of the tiebreaker option do not always clearly outline the conditions under which one can speak of a tiebreaker in organ allocation. Behind this concept, however, lies the general idea that there are prior, generally recognized and ethically far less questionable criteria, and that tiebreakers should only be used to bring about a decision if the primary criteria are not sufficient for this.

Brudney (2007) is one of the few authors who gives a more detailed description of the use of responsibility as a tiebreaker in liver allocation. He states a few conditions that must be met in order to use responsibility as a tiebreaker, namely that the ARESLD patient in question (in his example, the patient is named Jane) voluntarily and repeatedly put her health at risk by long-term heavy drinking (Brudney 2007, p. 42). Furthermore, he regards it as a necessary condition that the patients “know (or ought to know) basic facts about liver scarcity and transplant lists” (Brudney 2007, p. 45 f.). That is, patients should already be aware beforehand that alcohol-related liver diseases might lead to a lower priority on the transplant waiting list. Brudney acknowledges that the latter condition is, at the moment, rather rarely fulfilled. A group of patients that might actually meet this criterion are, according to Brudney, patients who need a re-transplantation:

“But I assume that the process of receiving a transplant is educative, and, in any event, it could be made educative. We could then assume that thereafter the agent knew enough or ought to have known enough. Thus if she comes to need a second transplant because of subsequent voluntary, health-risky conduct, it would be proper to sanction her by putting her lower, perhaps much lower, on the transplant list.” (Brudney 2007, p. 46).

Brudney describes tiebreaker situations as cases, in which an ARESLD patient has the same “chance of successful surgery and good long-term outcome” as “nonalcoholics on the list” (Brudney 2007, 42). “Suppose both Jane and Jack are candidates for the #1 slot and are essentially equal candidates, except that Jane’s liver disease is due to her alcoholism. (Brudney 2007, 44). “When information has been adequately distributed, Jane’s voluntary conduct becomes a form of callous disregard for and indifference to others’ dire needs. […] Under those circumstances, it is appropriate for a publicly funded institution to judge that Jane is morally less deserving than Jack of receiving that last liver.” (ibidem).

The condition that the candidates must be “essentially equal” is fundamental to the tiebreaker option. It is a shortcoming of the previous debate that most proponents of the tiebreaker option do not ask and explain in detail what it means that two patients are to be regarded as “essentially equal”. Of course, not every difference is relevant to the assessment of patients. It is implicitly assumed that only certain, namely ethically recognized criteria should be included in the assessment of the equality or inequality of patients. Thornton speaks of “similar clinical eligibility” (Thornton 2009, p. 739):

“Moreover, moral responsibility need only be used when two patients are otherwise equally eligible for one liver. If the alcohol-dependent individual is deemed most suitable when a liver becomes available for transplant then the criterion is not required. An alcohol-dependent individual’s history will therefore only be used as a tie-breaker.” (Thornton 2009, p. 740).

Although most proponents of the tiebreaker option do not explicitly discuss or specify which criteria are to be included in the assessment of the equality or inequality of patients, it can be assumed that they mainly think of the generally applied and recognized criteria of urgency and prospects of success which can be weighed and interpreted differently depending on the ethical approach one takes, and especially the MELD-score. However, there are international differences concerning which allocation criteria are deemed as ethically (and legally) permissible and should, hence, be included in the evaluation of the patients before any tiebreaker is used. For example, China generally takes six liver allocation factors into account, namely urgency (highly urgent patients are prioritized, all other patients ranked according to their MELD-score), geography, age (donor livers from patients < 12 years old are preferentially allocated to pediatric candidates that are less than 12 years old), blood type, the donor status of the patient or his/her family, and waiting time (National Health Commission of People’s Republic of China 2018). In contrast, the German liver allocation scheme is largely restricted to the criteria of urgency and prospects of success (in form of blood type matching), while waiting time is used as a form of tiebreaker. However, the German allocation scheme does not consider geographical factors or the donor status of the recipient and his/her family (Bundesärztekammer 2021).

It would be beyond the scope of the present paper to discuss which criteria should be considered before resorting to responsibility as a tiebreaker. Rather, we assume that there are several allocation criteria that, on a national level, are regarded as ethically and legally permissible for the allocation of the scarce resource of donor livers and are, hence, used as full-fledged allocation criteria. This generally includes the criteria of urgency and prospects of success, which can be weighted and interpreted differently. Furthermore, the MELD-score currently plays an important role in liver allocation, so that an allocation stalemate can occur especially when two patients have the same MELD score. These full-fledged criteria are used to rank patients, whereas a tiebreaker is only applied if, on the basis of the generally accepted criteria, several patients are to be considered equally suitable for a specific donor organ (allocation stalemate) in order to make a final allocation decision. It is obvious that the occurrence of a stalemate is all the more unlikely the more criteria are included in the allocation decisions, so that a tiebreaker would be needed relatively more often in the liver allocation system in Germany than, for example, in China. In general, it can be assumed that stalemate situations are very rare and rather unlikely to occur in clinical practice, especially if the waiting time is included as an allocation criterion, which can sometimes differ by a few days for patients with the same level of urgency and prospects of success. Regardless of the question of frequency, we will describe and analyze different kinds of scenarios, in which responsibility could be used as a tiebreaker.

### Different scenarios and ethical challenges of the use of responsibility as a tiebreaker

In light of the above-mentioned ethical challenges of the use of responsibility as an allocation criterion, four different scenarios should be differentiated in the context of the tiebreaker option, namely cases where a standoff arises (1) between ARESLD and non-ARESLD patients, (2) between several ARESLD-patients, (3) between ARESLD-patients where one of them needs a re-transplantation and the other one is waiting for his/her first transplantation, and (4) between ARESLD and non-ARESLD patients where the ARESLD-patient needs a re-transplantation and the non-ARESLD patient is waiting for his/her first transplantation. We will discuss these groups of cases individually.

In the first type of scenario, two patients are considered to be equally suitable for a donor organ, with the nationally applicable criteria for liver allocation being used in the assessment of their suitability. This scenario corresponds to the situation between Jane and Jack described by Brudney (2007) above. Although it seems nearly impossible to determine the exact degree of responsibility that an ARESLD patient has for the development of his/her liver failure, it has been argued by the proponents of the tiebreaker option (as seen above) that he/she did have at least some degree of control over his/her behavior. If an allocation stalemate occurs between this patient and another patient who is suffering from a life-threatening condition through no fault of him/herself (f. ex. the genetic Wilson disease), it can be regarded as justified and fair that responsibility is used as a tiebreaker in such a situation. Although this type of situations seems especially suitable for justifying the use of responsibility as a tiebreaker, a number of follow-up questions arise. There are several other liver diseases in which the influence of unhealthy behavior may play a causal role that is difficult to determine, so that the above-mentioned challenge of determining and comparing the degrees of responsibility of individual patients arises again. For example, nonalcoholic fatty liver disease (NAFLD) is a common indication for liver transplantation and may be linked to overweight or obesity (Shaker et al. 2014), just as hepatocellular carcinoma (HCC) may be linked to drug abuse, promiscuity, smoking, or overweight and obesity (Yang et al. 2019). This raises the ethically challenging question, why the ARESLD patients should be held accountable for their unhealthy behavior while others should not (Caplan 1994).

The problem of determining the level of responsibility becomes even more pressing in the face of the second type of situation in which two patients are considered to be equally suitable for a donor organ and both have been diagnosed with ARESLD. In such cases, it seems almost impossible to make an ethically informed decision in favor of one or the other patient based on the criterion of responsibility. In view of these concerns, it is necessary to examine whether, from an ethical point of view, other criteria, such as the waiting time, are better suited as tiebreakers in such cases – a question that will be briefly discussed in the next section.

Similar concerns can be raised regarding the third type of scenario, in which two ARESLD patients also compete for a particular donor liver. However, this third type of scenario differs from the second in some fundamental ethical aspects, which is why it is discussed as a separate group of situations. First of all, it has already been mentioned by Brudney that re-transplantation candidates can be assumed to have basic knowledge about the allocation process and the criteria used, as well as the life-threatening risks associated with continued alcohol consumption after their first transplant (Brudney 2007, p. 46). Thus, if an ARESLD patient needs a second transplant because of his/her sustained alcohol consumption it could be considered sufficiently justified to give preference to the equally suitable second patient who also has ARESLD but is awaiting his/her first transplant. It should be emphasized, however, that the relevant point here is the empirical reliability of our judgment of the two candidates’ knowledge of the effects of their alcohol consumption on their liver failure and a possible lower priority in the allocation process, not necessarily the actual knowledge of the patients. It could well be that the second patient, too, had sufficient knowledge of all these things in his/her previous life. However, this condition is rather rare and difficult to verify in the clinical context, which makes is ethically rather questionable to include such considerations in allocation decisions. Another ethically relevant aspect of the third type of stalemate situations is that the re-transplantation candidate has already received a share of the scarce life-sustaining resource of donor livers in form of his/her first transplantation. It can be argued that, for reasons of fairness, the second, equally suitable patient should also be given a corresponding share and, hence, be prioritized in similar stalemate situations (Dufner 2021, pp. 171 ff.).

These additional considerations of fairness and equal shares of a scarce resource may also be one of the reasons why the fourth type of cases, where a stalemate occurs between a non-ARESLD patient and an ARESLD-patient who needs a second transplant because of his/her sustained alcohol consumption, is seen as less ethically questionable and is even mentioned by Brudney as a paradigmatic use case for the criterion of responsibility. The fourth type of situations also seems to avoid the concerns expressed about the first type of allocation stalemates, namely that the non-ARESLD patients involved in the stalemate may also have some responsibility for their liver disease, such that the problem of determining and comparing the levels of responsibility arises. Just as in the third group of cases it can be argued that our judgment of the re-transplantation candidate’s knowledge of the health effects of his/her alcohol consumption and the liver allocation process can be considered as sufficiently justified, whereas this does not apply to the patient awaiting his/her first transplant.

As our discussion shows, responsibility is particularly suited as a tiebreaker in the third and fourth type of cases, while the second is rather inappropriate and the first type raises a number of considerable follow-up questions. The first and second groups of cases are particularly affected by ethical challenges in determining the level of causal control and responsibility that are linked to issues of equity and fairness. This was one of the key difficulties listed against the use of responsibility as a full-fledged allocation criterion and which the tiebreaker option was supposed to circumvent (see Sect. 2.3). In view of these concerns and the rather limited applicability of the criterion, the question arises whether there are not more suitable criterions that are confronted with fewer ethical concerns and could be used as a tiebreaker in liver allocation. In this context, the criterion of waiting time should be considered in particular.

### The use of waiting time as a tiebreaker: the better option?

Waiting time is a criterion commonly used as a tiebreaker in liver allocation, especially if two patients have a similar MELD-score (Veatch and Ross 2015, p. 316). Veatch proposes that responsibility should be used before waiting time as a tiebreaker (ibidem), although he does not offer an explicit discussion of the ethical arguments for/against the use of waiting time as a tiebreaker. The use of waiting time as an allocation criterion is closely linked to the general principle “first come, first served”. But why should the mere fact of waiting increase the legitimate claim of a person to a scarce and life-sustaining resource?

As one of the authors of this paper has pointed out in more detail elsewhere (Primc 2020b), the use of the waiting time as an allocation criterion is, in general, ethically justified in two different ways.^4^ The first line of justification points to desert-based principles which link the right to a good or resource (e.g. position, service, compensation, etc.) to individual merits or efforts: To each according to his/her contribution! As we wait, the proportion of the limited resource of time that we invest in obtaining a certain good increases. According to this interpretation, the “first come, first served” principle aims to achieve a fair balance between the waiting time invested (the costs) and the benefits derived from this investment. However, the principle “to each according to his/her contribution” can lead to an unfair distribution and increase of inequalities between individuals. This is particularly the case when individuals differ in their ability to make relevant contributions or efforts (Primc 2020b). In the context of waiting time and organ allocation, this is the case, for example, when patients suffer from liver diseases that progress at different rates, such that some patients ultimately have much less time at their disposal “to invest” in waiting for an organ. These concerns about the fairness of the waiting time criterion can be subsumed under the “moral luck” problem mentioned above, which generally describes the phenomenon that factors beyond our control may partially determine our moral praiseworthiness or blameworthiness (Hartman 2017, p. 23), i.e. our chance to get access to the scarce resource of donor livers.

The second line of justification interprets waiting time not as a substantive or material criterion of justice but rather as an aspect of procedural justice (Primc 2020b). According to Rawls, “pure procedural justice obtains when there is no independent criterion for the right result: instead there is a correct and fair procedure such that the outcome is likewise correct or fair” (Rawls 1971, p. 86). Since pure procedural justice defines the fairness of an allocation solely in terms of the correct application of a fair allocation procedure, even an extremely unequal distribution can be regarded as fair if everyone had equal opportunities to participate before the procedure was applied. Due to the numerous social and natural inequalities within society, the prerequisites of pure procedural justice are, however, rarely or never fulfilled in practice (ibidem). Social inequalities may influence access to health care as well as the general level of health literacy, i.e. the ability to access, understand, and communicate health care information, which includes the ability to navigate the health care system. This means that some patients may find it easier to get a referral to a transplant center, so they can access the waiting list faster than other patients who share the same level of urgency. Concerning natural inequalities, we can again refer to the uneven progression of liver diseases, which means that some groups of patients are able to wait longer for an organ than others, who, due to the nature of their disease or individual factors beyond their control, end up with final liver failure more quickly. Patients can fundamentally differ in their opportunity to wait for a donor organ, such that the prerequisites for a fair result are not fully met.

As this brief discussion shows, the criteria of responsibility and waiting time share some common disadvantages in terms of fairness and moral luck. However, a major advantage of the waiting time criterion is that it can be determined much more directly than the degree of a patient’s responsibility for his liver failure, since at least generally recognized units of measurement are available for the waiting time (e.g. days on the transplant list, or on the high-urgency list). A fundamental problem with regard to the criterion of responsibility is how to make it measurable and intersubjectively comparable. In addition, waiting time does not present the same ethical issues regarding the negative consequences of responsibility as an allocation criterion. Using responsibility as a tiebreaker would still entail the risk of negatively affecting the relationship between doctor and patient and that doctors will be pushed into the role of judges instead of advocates for their patients (Sharkey and Gillam 2010). Furthermore, it could increase the risk of stigmatization of patients suffering from alcohol disorders. “Stigmatization of people with mental disorders is a key contributor to healthcare inequality” (Kilian et al. 2021, p. 900). Several studies suggest that stigmatization of patients suffering from alcohol disorders is still a common phenomenon (Kilian et al. 2021; Room 2005; Schomerus et al. 2011, Arabaci et al. 2020). Kilian et al. (2021) identify precisely retrospective and prospective responsibility as key elements of the stigmatization process. Stigmatization can in turn lead to self-stigmatization of patients suffering from alcohol disorders (e.g. lack of confidence, loss of self-esteem, reduced self-efficacy) which may prevent them from seeking professional help in an early stage of their alcohol disorder and lead them to lying or withholding information about their history of alcoholism (Feiring 2008; Schomerus 2011).

Moreover, a possible negative consequence of the pervasive stigmatization of patients suffering from alcohol disorders is that doctors may be less willing to put them on the waiting list for liver transplantation. In a study on French doctors’ attitude towards patients suffering from ARESLD, Perut et al. (2009) suggest that the more unfavorable their attitude towards ARESLD-patients was, the less doctors were willing to grant them access to the scarce resource of donor livers. These negative effects are strong reasons for preferring the use of waiting time as a tiebreaker, even if this criterion is confronted with some equity concerns, too. In addition, we have argued that, from an ethical point of view, responsibility can only be applied in a very limited number of cases. In all other cases, another tiebreaker, such as the waiting time, would have to be used, so that the implementation of responsibility as a tiebreaker is of little benefit and comes with some considerable risks.

The benefits are further diminished if one considers that some allocation systems already include a larger number of criteria into their allocation decisions, such as the Chinese allocation scheme. As already mentioned above, China’s liver allocation policy generally uses six allocation factors: urgency, geography, age, blood type, donor status, and waiting time (National Health Commission of People’s Republic of China 2018). In this system, a donor liver from an adult donor would be allocated to the recipient with the highest MELD-score and who is located in the same hospital than the donor, who has been registered as a donor for more than three years, or whose main family members (spouse and relatives up to the third degree of kinship) have been organ donors themselves. If several candidates meet these criteria equally, waiting time and blood type matching will be considered to make an allocation decision. Hence, an allocation stalemate would only occur in China if all six factors are equal or at least very similar before the use of responsibility as a tiebreaker could be considered. We would tend to suggest that such cases are very few and, hence, the use of responsibility as a tiebreaker of even less benefit in similar allocation schemes.

## Conclusion

The present investigation has dealt with the question whether the tiebreaker option can serve as a compromise in the ethical debate surrounding the use of responsibility as an allocation criterion in liver transplantation. We identified two points of criticism that were given particular attention in the ethical debate, namely (1) empirical challenges in determining the individual degree of responsibility and the problem of moral luck, (2) concerns regarding the negative impact that the use of responsibility as an allocation criterion could have, namely on the relationship between doctors and their patients, as well as on the stigmatization of patients suffering from ARESLD. We identified four different types of situations in which responsibility could be used as a tiebreaker, namely cases where a standoff arises (1) between ARESLD and non-ARESLD patients, (2) between several ARESLD-patients, (3) between ARESLD-patients where one of them needs a re-transplantation and the other one is waiting for his/her first transplantation, and (4) between ARESLD and non-ARESLD patients where the ARESLD-patient needs a re-transplantation and the non-ARESLD patient is waiting for his/her first transplantation. We argued that responsibility is particularly suited as a tiebreaker in the third and fourth type of cases, while the first and second type of cases raises a number of ethical questions concerning the determination of the degree of responsibility and issues of moral luck – challenges that we already encountered in the responsibility debate and that the tiebreaker option was supposed to avoid. Furthermore, we have argued that the tiebreaker option does not address concerns about negative consequences, especially stigmatization of patients suffering from alcohol disorders such that other tiebreaker criterions, such as waiting time, should be preferred from an ethical point of view.

It could be objected that we ignore in our discussion other possible negative consequences that could arise from the fact that more and more ARESLD patients are being transplanted, e.g. that the general willingness to donate could decrease. This would lead to an overall lower number of donor organs. It should be emphasized that such a public reaction is precisely due to the stigmatization of ARESLD patients.

“The proper response to the problem of stigma is not to exclude patients who might benefit from transplants, but to educate the public about the importance of providing fair access to those in need and to call for the redoubling of efforts to find solutions to alcohol abuse.” (Caplan 1994, p. 221).

The debate surrounding the responsibility of ARESLD patients seems to focus on the wrong questions in other ways, too. Given the growing number of young adults getting waitlisted because of alcohol-associated liver disease, it would be more appropriate to focus the debate and calls for action on preventive measures to reduce the number of patients suffering from ARESLD and to make treatment of alcohol dependence and harmful alcohol consumption more readily accessible. As an increasing number of women are affected by alcohol dependence and harmful alcohol consumption, and as women (compared to men) tend to develop health problems with smaller amounts of alcohol, it has been argued that we need preventive and therapeutic approaches to alcohol addiction that are more specifically tailored to women (Philip et al. 2022; McCrady et al. 2020). Another approach to reducing the scarcity of donor livers is to increase the number of split liver transplants, i.e. to split every suitable donor liver so that two patients can be transplanted with one deceased donor liver. Split liver transplantation raises a number of additional ethical and medical questions that are beyond the scope of the present study and should be at the center of further ethical and medical research (Bobbert et al. 2019).

## Footnotes

1 For an overview of widely applied and recognized criteria in organ allocation, see Veatch and Ross (2015, pp. 284 ff.)

2 A comprehensive account of the debate has been given by Sharkey and Gillam (2010) as well as Notini et al. (2019).

3 Veatch himself seems to prefer a genuine tiebreaker option (Veatch and Ross 2015, p. 316).

4 We will not discuss lines of interpretation that justify waiting time as a proxy for urgency and/or prospects of success. The latter assume that the patients? state of health generally deteriorate over time, and with it usually their prospects of success. Waiting time is not seen here as an independent allocation criterion, but as an expression of urgency and prospects of success, which are generally accepted criteria in organ allocation. The central question in this context is how reliably the waiting time is able to reflect the urgency and/or prospects of success of individual patients.
